# Prognostic value of tumor-infiltrating lymphocytes and their association with clinical and pathological factors in gastric cancer

**DOI:** 10.1590/0102-672020260000019e1948

**Published:** 2026-07-27

**Authors:** Dhouha BACHA, Ines MALLEK, Mohamed HAJRI, Amani BENZARTI, Farah LOUED, Sana BEN-SLAMA, Lassad GHARBI, Ahlem LAHMAR

**Affiliations:** 1 University of Tunis, Faculty of Medicine - Tunis, Tunisia.; 2 University of Tunis El Manar, Faculty of Sciences of Tunis, Laboratory of Genetics, Immunology and Human Pathologies - Tunis, Tunisia.; 3 Mongi Slim University Hospital, Department of Pathology - Tunis, Tunisia.; 4 Mongi Slim University Hospital, Department of Surgery - Tunis, Tunisia.

**Keywords:** Adenocarcinoma, Prognosis, Survival, Lymphocytes, Tumor-Infiltrating, Stomach Neoplasms, Adenocarcinoma, Prognóstico, Sobrevivência, Linfócitos do Interstício Tumoral, Neoplasias Gástricas

## Abstract

**Background:**

The prognostic value of tumor-infiltrating lymphocytes has been studied in several cancers, but in gastric cancer, their evaluation by standard hematoxylin-eosin staining remains controversial.

**Aims:**

To analyze the prognostic value of tumor-infiltrating lymphocytes in gastric cancer and to investigate the association between tumor-infiltrating lymphocyte levels and a range of clinical and pathological factors in gastric cancer.

**Results:**

Forty-four patients were included with a mean age of 62 years, and 77% of the patients were classified as having pathological tumor stage 3 or pathological tumor stage 4. Lymph node metastases were noted in 66% of our patients. At diagnosis, 57% of patients were at an advanced stage (III). Patients with distant metastases were not included in this study. Evaluation of tumor-infiltrating lymphocytes in hematoxylin-eosin staining found that the mean tumor-infiltrating lymphocyte rate was 43 (extremes: 5-90%). 36% of patients had a tumor-infiltrating lymphocyte rate >50%. The overall survival of our patients was 40 months. Patients with tumor-infiltrating lymphocytes >50% had greater survival, without a significant correlation (p=0.275). A tumor-infiltrating lymphocyte rate =50% was correlated with age >50 years (p=0.030), presence of lymph node involvement (p=0.023), and advanced tumor stage (III+IV) (p=0.05). In a multivariate analysis, the presence of lymph node involvement (p=0.045) and advanced tumor stage (III+IV) (p=0.015) were identified as independent factors associated with a low tumor-infiltrating lymphocyte rate =50%.

**Conclusions:**

Poor prognostic factors for gastric cancer (age >50 years, presence of lymph node involvement, and advanced tumor stage) were associated with a low tumor-infiltrating lymphocyte rate assessed by hematoxylin-eosin staining. However, standardization of tumor-infiltrating lymphocyte assessment is essential to allow comparability between studies and integrate this parameter into routine practice.

## ARTICLE HIGHLIGHTS

• Stromal tumor-infiltrating lymphocytes were assessed on routine hematoxylin-eosin slides in 44 surgically treated patients with non-metastatic gastric adenocarcinoma.

• Patients with tumor-infiltrating lymphocytes >50% showed longer overall survival, although the difference was not statistically significant.

• Low tumor-infiltrating lymphocyte rates were significantly associated with age >50 years, lymph node involvement, and advanced tumor stage.

• Lymph node involvement and advanced tumor stage were independent factors associated with low tumor-infiltrating lymphocyte rates, supporting the potential prognostic value of tumor-infiltrating lymphocytes in gastric cancer.

## Summary

**CENTRAL MESSAGE:**

The study of the inflammatory microenvironment has emerged as one of the most promising avenues of research to better understand the role of the immune system in tumor progression. This has led to the identification of new factors, especially tumor-infiltrating lymphocytes. Their prognostic value has been studied in other cancers (such as breast cancer), but in gastric cancer, the prognostic value of tumor-infiltrating lymphocytes assessed by standard hematoxylin-eosin staining remains controversial, partly because of the lack of consensus on how this parameter should be evaluated.

**PERSPECTIVES:**

The consideration of tumor-infiltrating lymphocytes in gastric cancer holds promise for understanding immune interactions in the tumor microenvironment, prognostic assessment, and the use of immunotherapy. Our study suggests that tumor-infiltrating lymphocytes assessed by hematoxylin-eosin staining in patients with gastric cancer may be a prognostic indicator on a par with already established clinical-pathological parameters. Advances in digital pathology and artificial intelligence also offer possibilities of automation for more precise quantification of tumor-infiltrating lymphocytes.

## VISUAL ABSTRACT

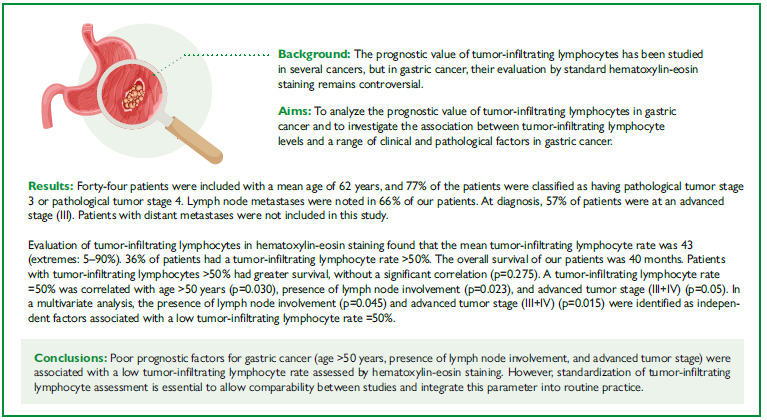

## RESUMO

**Racional:**

O valor prognóstico dos linfócitos infiltrantes tumorais (LITs) foi estudado em vários tipos de câncer, mas no câncer gástrico (CG), sua avaliação por coloração padrão de hematoxilina-eosina (HE) continua controversa.

**Objetivos:**

Analisar o valor prognóstico dos LITs no CG e investigar a associação entre os seus níveis e diversos fatores clínicos e patológicos.

**Resultados:**

Foram incluídos 44 pacientes com média de idade de 62 anos, sendo que 77% foram classificados como pT3 ou pT4. Metástases linfonodais foram observadas em 66% dos pacientes. 57% estavam em estágio avançado (III) no diagnóstico. Pacientes com metástases à distância não foram incluídos. A avaliação dos LITs por coloração em hematoxilina-eosina mostrou uma taxa média de 43% (extremos: 5-90%), sendo que 36% apresentaram LITs >50%. A sobrevida global foi de 40 meses. Pacientes com LITs >50% tiveram sobrevida maior, sem significância estatística (p=0,275). Uma taxa de LITs=50% foi associada à idade >50 anos (p=0,030), presença de acometimento linfonodal (p=0,023) e estágio tumoral avançado (III+IV) (p=0,05). Na análise multivariada, a presença de acometimento linfonodal (p=0,045) e o estágio tumoral avançado (III+IV) (p=0,015) foram identificados como fatores independentes associados a uma baixa taxa de LITs=50%.

**Conclusões:**

Fatores prognósticos desfavoráveis no CG (idade >50 anos, envolvimento linfonodal e estágio tumoral avançado) foram associados a uma baixa taxa de LITs avaliada em HE. No entanto, é essencial padronizar a avaliação dos LITs para permitir a comparabilidade entre estudos e integrar esse parâmetro à prática clínica.

## INTRODUCTION

Gastric cancer (GC) is the fifth most common type of cancer worldwide and the fourth leading cause of cancer-related deaths^21^. Despite the development of numerous therapeutic modalities, the 5-year survival rate for all stages combined is around 20%, and almost 50% of patients develop a local or distant recurrence^24^. The study of the inflammatory microenvironment has emerged as one of the most promising avenues of research to better understand the role of the immune system in tumor progression^1^ ^,^ ^16^. This has led to the identification of new factors, especially tumor-infiltrating lymphocytes (TILs). Their prognostic value has been studied in other cancers, such as breast cancer^19^, but in GC, the prognostic value of TILs assessed by standard hematoxylin-eosin (HE) staining remains controversial, partly because of the lack of consensus on how this parameter should be evaluated.

Our study aimed to clarify the prognostic value of TILs in GC through a survival study and to evaluate the correlation between TIL density and a set of clinical-pathological factors in GC.

## METHODS

### Study design

This study was a retrospective and longitudinal analysis of a single-center series of patients with gastric adenocarcinoma who underwent surgery in the general surgery department. All cases were collected from the Department of Pathology of the same hospital, from January 2008 to December 2023.

### Ethics and consent

The Ethics Committee for Mongi Slim University Hospital (La Marsa, Tunisia) has examined the study and protocol of the following project: “TILs in gastric cancer” (number 042024). The project does not raise any particular ethical problem. Due to the retrospective nature of this data collection study, no prior consent was required from patients. Data anonymity was respected.

### Study population

We included patients with non-metastatic gastric adenocarcinoma treated in the General Surgery department. We excluded patients who had not undergone surgery, cases with only gastric biopsies available, other malignant tumors that were not adenocarcinomas (other carcinomas, lymphomas, stromal tumors, and neuroendocrine tumors), and patients who had received neoadjuvant chemotherapy. Additionally, patients whose hospital records were unusable or could not be found, and cases with non-usable slides or tissue blocks were excluded from the study.

### Data collection

We collected epidemiological, clinical, and biological data, as well as the type of surgical procedure performed, follow-up, and outcome for all patients included in the study.

### Pathological study

The macroscopic data were collected from pathological reports, including the type of specimen received, tumor location, tumor appearance, and size.

Two senior pathologists reexamined the HE-stained slides.

The following histological parameters were recorded: the degree of differentiation of the adenocarcinoma, the presence or absence of vascular emboli, perineural invasion, the lymph node status, and the presence of associated lesions.

We applied the Lauren classification^7^, World Health Organization (WHO) classifications^12^, and the 8th edition of the Union for International Cancer Control (UICC) pTNM (pathological tumor, lymph node, and metastasis) Classification, published in 2017^24^.

### Pathological study of tumor-infiltrating lymphocytes

We examined an HE-stained slide on a representative block per tumor to quantify TILs in the carcinomatous stroma. We used the recommendations of the International Working Group on TILs, which appeared in 2014^2^ ^,^ ^18^.

### Statistical analysis

To investigate the correlation between prognostic factors and TIL rate, continuous variables were compared using the non-parametric Mann-Whitney U test. Ordinal variables were compared using a chi-squared test. Logistic regression was used to identify prognostic factors. All statistical tests were performed at a significance level of 5%.

## RESULTS

### Descriptive study

We included 44 patients. The mean age of our patients was 62 years±15, with a median of 66 years (extremes: 24-85). Our population comprised 30 males (68%) and 14 females (31%). Sex ratio (male/female) was 2.14. In 61% of cases, risk factors associated with GC were observed. Smoking was the most frequent one in 47% of cases, followed by chronic atrophic *Helicobacter pylori* gastritis in 40%. In addition, 14% of patients had gastric ulcers.

The mean time to diagnosis was 7.1 months. General signs (asthenia, anorexia, weight loss) were noted at the discovery of GC in 55% of patients. The most frequent functional sign was epigastralgia (36 patients, 82%). All patients underwent esophagogastroduodenal fibroscopy, which revealed that the GC was located in the fundus in 55% of cases, in the antrum in 39%, in the cardia in two patients, and was pan-gastric in one case. The predominant macroscopic appearance of the tumor was ulcerative-bourgeous in 89% of cases. Polypoid mass was present in two patients, as was ulceration (4%). Gastric infiltrative appearance was noted in one patient. The tumor was stenosing in three cases (7% of cases). All patients underwent thoracic-abdominal-pelvic computed tomography, confirming the absence of distant metastases. Therapeutic management was a gastrectomy in all patients. Lymph node dissection was D1.5 in 73% of cases and D2 in 27%.

### Pathological study

The surgical specimens consisted of total gastrectomies in 66% of patients and partial gastrectomies in 34%. The mean tumor size was 55 mm±24 mm (extremes: 15 to 130 mm). Tumor size larger than 50 mm was found in 45% of patients. Based on Lauren’s classification, adenocarcinomas were intestinal in 17 patients (39%), diffuse in 22 (50%), and mixed in five (11%). According to the WHO 2019 classification, GC was a poorly cohesive carcinoma in 22 patients (50%), tubular in 13 patients (29%), mixed in five patients (11%), papillary in two patients (5%), and mucinous in two patients. It was also low-grade or well-differentiated in 20 patients (45%), high-grade in 24 patients (55%), moderately differentiated in 14 patients, and poorly differentiated in 10 patients. Vascular emboli were noted in 43% of cases. Perineural invasion was observed in 43% of cases. Surgical margins were healthy (R0) in 39 cases (89%) and microscopically tumoral (R1) in the remaining five. Histological lesions were noted in 80% of patients (35 cases), related to *Helicobacter pylori* infection in 25 patients. For pTNM staging, 77% of patients (34 cases) were classified as having pathological tumor 3 (pT3) or pT4. Two were classified as pT1 and eight as pT2. For N status, lymph node metastases were noted in 29 cases (66%). At diagnosis, 57% of patients were at an advanced stage (III). Patients with distant metastases were not included in this study.

### Tumor-infiltrating lymphocyte assessment

**Figure 1.**
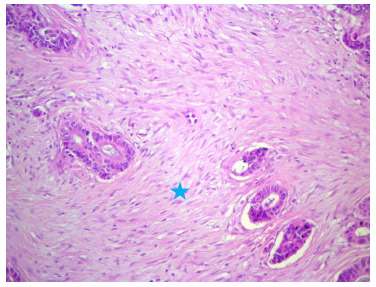
Well-differentiated tubular adenocarcinoma with fibrous stroma and a low tumor-infiltrating lymphocytes rate (Hemato-Eosin X 25).

Source: Personal archive.

**Figure 2.**
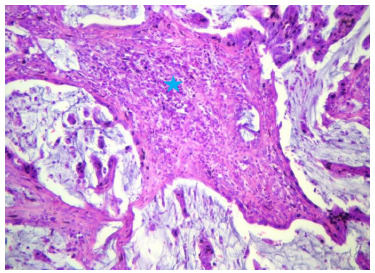
Mucinous adenocarcinoma with tumor-infiltrating lymphocytes evaluated at 45% (star) (Hemato-Eosin X 40).

Source: Personal archive.

**Figure 3.**
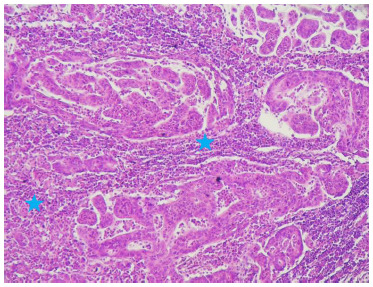
Moderately differentiated tubular adenocarcinoma with tumor-infiltrating lymphocytes evaluated at 80% (Stars) (Hemato-Eosin X 25).

Source: Personal archive.

TIL rates ranged from 5% to 90%, with a mean of 43% and a median of 40%. Sixteen patients (36%) had a TIL rate between 51 and 100% (Figure 1, Figure 2, and Figure 3).

### Analytical study

The overall survival (OS) of our patients was 40 months. Median OS for the entire population was 36 months (extremes: 0 and 130 months). As patients lost to follow-up were counted as deceased, there were a total of 19 deaths (43%) and 25 survivors up to December 2023.

In our series, univariate analysis showed that the factors associated with better OS were intestinal histological type according to Lauren’s classification^7^, well-differentiated gastric adenocarcinoma, absence of vascular emboli, and GC stage I or II. Patients with a TIL rate above 50% had a greater OS with no significant difference (48 months vs. 41 months, p=0.275) (Table 1).

**Table 1.** Analysis of different prognostic factors in relation to survival.

Variables	Total n (%)	Median survival	p-value
**Demographics**			
Age (year)			
>50	33 (75)	41	0.723
=50	11 (25)	40	
Gender			
Male	30 (68)	39	0.355
Female	14 (32)	40	
Related to primary GC			
Risk factors of GC			
Present	27 (61)	40	0.417
Absent	17 (39)	42	
General signs			
Present	24 (55)	40	0.121
Absent	20 (45)	44	
Site of GC			
Fundus	24 (55)	48	0.877
Other	20 (45)	41	
**Pathological**			
Resection type			
Total gastrectomy	29 (66)	41	0.648
Partial gastrectomy	15 (34)	48	
Tumor size (mm)			
=50	24 (55)	41	0.707
>50	20 (45)	44	
Histological type/Lauren classification^7^			
Intestinal	17 (39)	109	0.044
Diffuse	22 (50)	41	
Mixed	5 (11)	36	
Histological type/WHO classification			
Tubular	13 (29)	70	0.159
Other	31 (71)	41	
Well differentiated			
Yes	20 (45)	50	0.004
No	24 (55)	38	
Vascular emboli			
Present	19 (43)	40	0.05
Absent	25 (57)	48	
Perineural invasion			
Present	19 (43)	48	0.806
Absent	25 (57)	38	
Surgical margins			
R0	39 (89)	41	0.791
R1	5 (11)	70	
pT			
pT3+pT4	34 (77)	42	0.928
pT1+pT2	10 (23)	39	
pN			
N0	15 (34)	48	0.925
N+	29 (66)	41	
Stage			
I+II	19 (43)	84	0.001
III	25 (57)	37	
TILs			
=50%	28 (64)	41	0.275
>50%	16 (36)	48	

GC: gastric cancer; WHO: World Health Organization; pT: pathological tumor; pN: pathological lymph node; TILs: tumor-infiltrating lymphocytes; mm: millimeter.

In our series, a TIL rate =50% was correlated with age >50 years (p=0.030), presence of lymph node invasion (pTN+) (p=0.023), and advanced tumor stage (stage III+IV) (p=0.05). A rate of TILs =50% was more frequent in the male gender, presence of risk factors of GC, presence of general signs, fundal location of GC, gastrectomy resection, tumor size >50 mm, Lauren’s intestinal histological type, WHO tubular histological type, poor differentiation, presence of vascular emboli, invaded margins (R1), advanced pT (pT3 + pT4), and the occurrence of death, but with no significant relationship (Table 2).

**Table 2.** Correlation between tumor-infiltrating lymphocytes and other prognostic factors.

Variables	TILs=50% n (%)	TILs >50% n (%)	p-value
Demographics			
Age >50 years	18 (41)	15 (34)	0.030
Male gender	19 (43)	11 (25)	0.612
Related to primary GC			
Presence of CG risk factors	17 (39)	10 (22)	0.583
Presence of general signs	16 (37)	8 (18)	0.442
Fundal site of GC	16 (37)	8 (18)	0.442
Pathological			
Resection: total gastrectomy	20 (46)	9 (20)	0.244
Tumor size >50mm	15 (34)	5 (11)	0.132
Histological type Intestinal/Lauren classification^7^	11 (25)	6 (14)	0.676
Histological type Tubular/WHO	10 (23)	3 (6)	0.442
Poor differentiation	18 (41)	6 (13)	0.080
Presence of vascular emboli	12 (27)	7 (16)	0.6
Presence of perineural invasion	14 (32)	5 (11)	0.187
Surgical margins R1	4 (9)	1 (2)	0.392
pT: pT3+pT4	23 (53)	11 (25)	0.256
pN: N+	22 (50)	7 (16)	0.023
Stage: III+IV	19 (43)	6 (14)	0.05
Outcome: death	14 (32)	5 (11)	0.187

GC: gastric cancer; WHO: World Health Organization; pT: pathological tumor; pN: pathological lymph node; TILs: tumor-infiltrating lymphocytes; mm: millimeter.

In a multivariate analysis, after adjustment for confounding variables, among the three significant variables identified in univariate analysis, the presence of lymph node involvement (pN+) and advanced tumor stage (III) in operated GC specimens were identified as independent factors associated with a low TIL rate =50%. In fact, a low TIL rate was correlated with age over 50 (odds ratio (OR) =0,31; information coefficient 95%CI 0.20-0.81; p=0.380), lymph node invasion (N+) (OR 3.52; 95%CI 1.13-6.94; p=0.045), and an advanced tumor stage (III) (OR 2.34, 95%CI 1.36-8.08; p=0.015).

## DISCUSSION

In our study, we evaluated the prognostic value of TILs in a cohort of 44 patients who had undergone GC surgery.

Classical prognostic factors associated with better survival in GC were partially confirmed, including the intestinal histological type according to Lauren’s classification^7^, well-differentiated adenocarcinoma, absence of vascular emboli, and early tumor stage (I or II).

Few studies have evaluated the correlation between TILs assessed on HE slides, survival, and prognostic factors in GC.

With the rise of immunotherapy, interest in TILs has been renewed across several cancers, including colorectal, lung, ovarian, and breast cancer^2^ ^,^ ^6^ ^,^ ^19^ ^,^ ^26^.

The TIL rate corresponds to the ratio of the surface area occupied by mononuclear inflammatory stromal cells to the total surface area of the tumor stroma.

A distinction is made among intratumoral TILs, which are mononuclear cells in direct contact with tumor cells; stromal TILs, which are in the immediate tumor cell environment, but not in direct contact with it; and lymphocytes forming tertiary lymphoid structures within tumors. These structures are composed of a zone of T lymphocytes in contact with a follicular zone of B lymphocytes, and the presence of a germinal center.

The assessment of TILs is generally treated as a continuous variable, expressed as a percentage within the observation zone. However, in some studies, it can be considered as a binary variable, distinguishing between high and low levels^6^.

Several histological and immunohistochemical approaches to the use of flow cytometry and various automata have been employed to quantify TILs in breast and other cancers. The International Working Group on TILs has proposed guidelines, initially for breast cancer, which have since been adapted to other tumor types^2^ ^,^ ^18^. The evaluation of TILs by this method, because of its simplicity and subjective nature, has raised questions about the reliability and reproducibility of its results.

Despite concerns about interobserver variability, several studies have reported acceptable reproducibility, particularly for stromal TILs^6^ ^,^ ^23^.

Immunoscore, an immunohistochemistry (IHC)-based tool predominantly used in colorectal cancer, quantifies Cluster of Differentiation 3-positive (CD3+) and Cluster of Differentiation 8-positive (CD8+) T cells and has been proposed as a complementary biomarker to the TNM (tumor, nodes, and metastasis) classification^3^ ^,^ ^13^ ^,^ ^26^.

TILs reflect the host’s immune response to cancers and are associated with the prognosis of various solid cancers. In breast cancer, for example, high levels of TILs significantly increase the sensitivity and efficacy of neoadjuvant chemotherapy^27^. In colon cancer, TILs are a well-confirmed prognostic parameter for localized cancer^17^.

In our study, evaluation of TILs in HE found that the TIL rate ranged from 5 to 90%, with a mean of 43% and a median of 40%. Sixteen patients (36%) had a TIL rate exceeding 51%.

The OS of our patients was 40 months (extremes: 0 and 130 months). Patients with TILs above 50% had longer OS, with no significant difference (48 months vs. 41 months, p=0.275). Factors associated with better OS were intestinal histological type according to Lauren’s classification^7^, well-differentiated nature of gastric adenocarcinoma, absence of vascular emboli, and GC stages I+II.

A TIL rate =50% was significantly correlated with age over 50 years (p=0.030), lymph node involvement (pTN+) (p=0.023), and advanced tumor stage (III) (p=0.05). In a multivariate analysis, lymph node involvement (pN+) and advanced tumor stage (III) in operated GC specimens were identified as independent factors associated with a low TIL rate =50% (OR 3.52, p=0.045 and OR 2.34, p=0.015, respectively).

Our findings concur with those of Tian et al.^22^, who demonstrated that high TILs were associated with greater survival compared with low TILs (p=0.003). These results indicated that a high TIL rate assessed on HE may provide important information regarding the survival of patients with GC. To our knowledge, this is the only meta-analysis to have studied the association between TILs assessed by HE and survival in GC patients. Moreover, our results are in line with those of meta-analyses that have studied the prognostic value of TILs assessed by IHC in GC. Thus, high levels of CD8+, CD3+, and CD4+ T-cell infiltration in tumor tissue were significantly associated with improved survival in GC patients^5^ ^,^ ^9^ ^,^ ^28^ ^,^ ^29^.

A recent study by Pereira et al.^14^ further supports these findings. In a large cohort of 345 patients, high CD3+ TIL density, quantified by IHC, was independently associated with superior OS (Hazard Ratio (HR)=1.51, p=0.018). Notably, high CD3+ infiltration was correlated with Epstein-Barr virus (EBV)-positive and PD-L1(Programmed Death-Ligand 1)-positive tumors, therefore, underlining the immunologic heterogeneity of GC and the potential utility of TILs as biomarkers for immunotherapy stratification.

However, data from a 2023 meta-analysis by Ren et al.^15^ challenge this consensus. Including 11 studies and nearly 2,300 patients, the authors found no statistically significant association between TIL density and OS (HR 0.95, 95%CI 0.62-1.45). The authors highlighted methodological heterogeneity and variation in TIL definitions as potential reasons for these discrepancies. These contrasting findings underscore the urgent need for standardized protocols in TIL evaluation.

Finally, our result is consistent with previous studies on other cancers: high TIL density has been recognized as an independent prognostic marker for good OS^4^ ^,^ ^8^ ^,^ ^13^ ^,^ ^27^.

The meta-analysis by Tian et al.^22^ included 2,835 cases from nine of the observational studies, demonstrating that a high density of TILs was significantly associated with favorable clinical and pathological factors: a lower degree of invasion (T1-T2 vs. T3-T4; p<0.001), absence of lymph node involvement (presence vs. absence; p<0.001), and earlier TNM stage (III-IV vs. I-II; p<0.001). However, TIL density was not correlated with age, gender, Lauren classification^7^, or histological grade. A retrospective study by Zhang et al.^25^ showed that high TIL levels were significantly correlated with small tumor size (p<0.001), well-differentiated carcinoma (p<0.001), absence of lymph node metastases (p<0.001), absence of nerve invasion (p<0.001), absence of tumor thrombosis (p=0.003), early/low pTN stage (p<0.001), and radical gastrectomy (p<0.001). Currently, in addition to being prognostic biomarkers for GC, TILs are potential targets for immunotherapy. Therapies that stimulate the immune system, such as immune checkpoint inhibitors (e.g., PD-1/PD-L1 inhibitors), may be more effective in patients with high lymphocyte infiltration for unresectable locally advanced tumors and metastatic forms. In fact, several studies have validated the value of TILs as a predictor of treatment response, OS, and relapse-free survival to integrate this parameter into the new TNM-Immune classification proposed. In the phase II KEYNOTE-158 study, 24 GC patients were treated with the humanized anti-PD1 monoclonal antibody pembrolizumab, and 11 patients showed responses with a median progression-free survival of 11 months^11^. In the phase III KEYNOTE-062 trial, pembrolizumab anti-PD1 monotherapy had similar OS to chemotherapy with fluoropyrimidine plus cisplatin.

However, progression-free survival was shorter^20^. In addition, higher TIL levels were associated with better chemotherapy efficacy. Stage II-III GC patients treated with the FOLFOX (oxaliplatin+leucovorin+fluorouracil) protocol with high TILs had better OS^10^. Overall, the therapeutic benefit of TILs, in this case, a high level of TILs, is twofold: to potentiate the effect of both chemotherapy and immunotherapy in advanced GC.

All in all, our findings support the relationship between a high density of TILs in GC and certain prognostic factors that have already been confirmed, and suggest that age >50 years may also be linked to lower TIL levels.

The main limitation of our study is, probably, its retrospective nature, the relatively small number of cases, which may limit statistical study groups, and the lack of standardization of macroscopic protocols (tumor sampling).

## CONCLUSIONS

The consideration of TILs in GC holds promise for understanding immune interactions in the tumor microenvironment, prognostic assessment, and the use of immunotherapy. Our study suggests that TILs assessed by HE staining in patients with GC may be a prognostic indicator on a par with already established clinical-pathological parameters. To date, there is no consensus on HE-assessed TILs in GC, as there is in breast cancer. Advances in digital pathology and artificial intelligence also offer automation possibilities for more precise quantification of TILs.

## Data Availability

The datasets generated and/or analyzed during the current study are available from the corresponding author upon reasonable request.
